# Factors Associated with Pregnancy Success and Calf Growth via Multiple Ovulation and Embryo Transfer in Holstein–Friesian Cattle

**DOI:** 10.3390/ani16172694

**Published:** 2026-08-30

**Authors:** Eszter Tóth, János Posta, Viktor Szőnyi, József Rátky, Renáta Knop

**Affiliations:** 1 Doctoral School of Animal Science, University of Debrecen, H-4032 Debrecen, Hungary; 2 Department of Animal Science, Institute of Animal Science, Biotechnology and Nature Conservation, Faculty of Agricultural and Food Sciences and Environmental Management, University of Debrecen, H-4032 Debrecen, Hungary; 3 Sz-Consult Ltd., H-4080 Hajdúnánás, Hungary; 4 Department of Obstetrics and Farm Animal Medicine Clinic, University of Veterinary Science, István Street 2, H-1078 Budapest, Hungary

**Keywords:** embryo transfer, Holstein–Friesian, pregnancy rate, term calving rate, post-diagnosis gestation loss, fresh embryos, frozen–thawed embryos, birth weight

## Abstract

This study evaluated factors affecting pregnancy outcomes, full-term calving success, and pre-weaning performance following embryo transfer in Holstein–Friesian cattle under commercial farm conditions. A total of 604 in vivo-derived embryos (fresh and frozen–thawed) were transferred into 384 heifers and 220 cows, and pregnancies were diagnosed by ultrasonography. Calves were monitored from birth to weaning. Pregnancy rate per embryo transfer (PR/ET) was higher in heifers than in cows, indicating superior reproductive performance of heifer recipients under field conditions. Recipient corpus luteum (CL) stage II yielded favorable pregnancy outcomes compared to CL stage I, whereas CL stage III comprised a small subgroup (*n* = 8) exhibiting a descriptive PR/ET of 87.5% that required cautious interpretation due to the limited sample size. Fresh embryos were associated with higher PR/ET than frozen–thawed embryos. Embryo developmental stage did not significantly affect initial pregnancy establishment or full-term calving rates, although stage 6 embryos (blastocyst) showed a non-significant numerical trend toward higher calving rates. Post-diagnosis gestation loss was a primary limiting factor, particularly in multiparous cows. Regarding pre-weaning performance, calves derived from frozen–thawed embryos exhibited a slightly higher birth weights than those from fresh embryos, whereas weaning weight and pre-weaning average daily gain did not differ between embryo types. Overall, recipient category and embryo type are key factors influencing pregnancy success, whereas postnatal performance is less affected. These findings highlight the importance of recipient selection and embryo management strategies for improving reproductive efficiency under commercial dairy farm conditions.

## Simple Summary

Embryo transfer is widely used in dairy cattle breeding to produce offspring from genetically superior animals, but its success depends on several factors. This study investigated how the type of embryo used and the characteristics of the female receiving the embryo influenced pregnancy success and calf growth under commercial dairy farm conditions. We found that young females that had not yet calved became pregnant more often than cows that had already produced calves. Fresh embryos were also associated with higher pregnancy rates per embryo transfer than frozen and thawed embryos. In contrast, the stage of embryo development had little influence on the chance of establishing a pregnancy. Calves born from frozen and thawed embryos were slightly heavier at birth than those born from fresh embryos, but both groups grew at a similar rate until weaning. These results show that selecting suitable recipient animals and using fresh embryos whenever possible can improve the success of embryo transfer. The findings provide practical information for dairy farmers and breeding programs aiming to increase reproductive efficiency and make better use of valuable genetics under commercial farming conditions.

## 1. Introduction

Among assisted reproductive technologies (ARTs), multiple ovulation and embryo transfer (MOET) is a long-established and widely implemented method in cattle breeding. Artificial insemination (AI), particularly when utilizing semen from genetically superior sires, has enabled substantial genetic progress across dairy herds. Furthermore, the widespread adoption of sex-sorted semen has enhanced breeding efficiency by facilitating the targeted production of replacement heifers and enabling the expanded use of beef crossbreeding [1,2,3]. While the integration of these reproductive technologies accelerates genetic gain and improves herd management flexibility, the successful application of ART ultimately depends on balancing reproductive efficiency with the long-term health and development of the offspring.

In the MOET programs, embryos develop in vivo, which provides a more favorable physiological environment for early embryonic development than in vitro systems. Nevertheless, evaluating birth outcomes, particularly calf birth weight, remains essential. Although the global proportion of in vitro-produced embryos continues to rise [4], traditional in vivo techniques remain fundamental to commercial livestock breeding. Consequently, assessing offspring performance is a critical component in evaluating the long-term efficacy and biological implications of ART applications.

The production of healthy offspring is a fundamental prerequisite for the widespread application of ART. While developmental abnormalities are most commonly associated with in vitro systems, variation in birth weight also occurs in in vivo-derived embryos. This has significant practical implications, as elevated birth weight is a primary risk factor for dystocia [5,6], which can compromise both the dam and calf health [7]. Consequently, identifying the determinants of calf birth weight is of particular importance, as early-life traits directly influence neonatal survival, pre-weaning growth rate, and future productive lifetime efficiency.

The commercial application of frozen–thawed embryo transfer commenced in the early 1980s and advanced substantially throughout in the 1990s following the introduction of ethylene glycol as a cryoprotectant to replace glycerol [8,9]. This breakthrough facilitated the direct transfer of thawed embryos under field conditions. eliminating the requirement for post-thaw microscopic evaluation and complex step-down washing procedures. However, cryopreservation exposes embryos to various physical and physiological stressors that may compromise viability and reduce pregnancy rates [10,11]. Despite these technological advances, embryo cryopreservation remains a delicate biological process during which embryos experience osmotic, thermal, and chemical stress that can influence their subsequent developmental competence. Consequently, both embryo stage and morphological quality are known to influence pregnancy success. Because frozen–thawed embryos play a crucial role in breeding programs and calving synchronization, selecting embryos based on optimal developmental stage is of particular importance. Maintaining pregnancy and achieving full-term calving success after transfer are critical determinants of reproductive efficiency and farm profitability. Post-diagnosis pregnancy loss and abortion diminish overall success rates and prolong the calving-to-conception interval, leading to increased production costs and economic losses [12,13,14]. Therefore, monitoring pregnancy outcomes in relation to embryo characteristics is essential. In addition to embryo-related factors, recipient characteristics, such as age category and corpus luteum status, may also influence uterine receptivity and pregnancy maintenance following transfer.

Previous studies on cryopreservation have reported that offspring derived from frozen–thawed embryos may exhibit altered birth weights under certain conditions [15,16,17]. However, a clear distinction must be made regarding embryo origin: while pronounced fetal overgrowth and major epigenetic alterations (such as Large Offspring Syndrome) are predominantly associated with in vitro-produced (IVP) or cloned embryos, the effects on in vivo-derived embryos are considerably more subtle [18]. Specifically, the maternal endocrine environment and placentation dynamics play pivotal roles in regulating fetal growth. In in vivo-derived embryos, sub-lethal thermal and osmotic stress induced specifically by the cryopreservation process itself may cause transient alterations in gene expression or placental adaptive mechanisms, which may contribute to minor downstream phenotypic variations in postnatal development without the severe anomalies observed in IVP systems [18].

Advances in cryopreservation techniques have substantially improved post-thaw embryo viability and developmental competence, driving the widespread adoption of this technology [9]. Furthermore, differences between fresh and frozen–thawed embryos may influence birth outcomes, although these effects are generally modest [19]. 

Therefore, the objective of this study was to evaluate the effects of recipient category (heifer or cow), recipient corpus luteum (CL) stage (I-III), embryo type (fresh or frozen–thawed), and embryo developmental stage (stages 4–7) on pregnancy rates in Holstein–Friesian cattle. In addition, the influence of these factors on calf performance, specifically birth weight, weaning weight, and average daily gain during the pre-weaning period, was assessed.

## 2. Materials and Methods

### 2.1. Study Design and Animals

This study was conducted under commercial on-farm conditions on a Holstein–Friesian dairy farm in Hungary maintaining an average total herd size of approximately 1500 cows, with embryo recovery and transfer procedures executed on a routine monthly schedule between 2022 and 2025. All animals were housed in loose housing systems. The facilities provided well-ventilated, pavilion-style structures; ad libitum access to water and total mixed ration (TMR); and automated feed delivery and manure removal. Donor heifers represented approximately 10% of the herd and were selected using commercial genomic evaluation, allowing embryo collection from as early as 11–12 months of age. Monthly cohorts of replacement heifers reaching 11–12 months of age underwent commercial genomic testing: heifers ranking in the top 5–10% for genomic merit were assigned as embryo donors, whereas the remaining herd-matched heifers entered the synchronization program as potential recipients. A total of 604 embryo transfers were performed during the study period. Recipient animals included 384 heifers and 220 cows. Each recipient animal was utilized only once during the study period, establishing zero recipient reuse across transfers. Recipient selection adhered to strict health and reproductive criteria: animals were certified as free from major infectious reproductive pathogens (BVDV, IBR, Brucellosis), maintained a body condition score (BCS) between 2.75 and 3.50 (1–5 scale), and exhibited normal pubertal/postpartum reproductive tract morphology without clinical endometritis or abnormal discharge. The transferred embryos originated from 119 unique donor heifers (mean ± SE = 5.08 ± 0.38 transferred embryos per donor; range: 1–23) bred using semen from 86 unique sires. Pregnancy outcomes were recorded, and calves born from these transfers (*n* = 171) were monitored from birth until weaning between 2023 and 2026. To ensure operational consistency across years and eliminate operator bias, all embryo recoveries, morphological evaluations, and non-surgical transfers were performed by a single certified veterinarian using standardized techniques.

### 2.2. Superovulation of Donor Heifers

**Figure 1:**
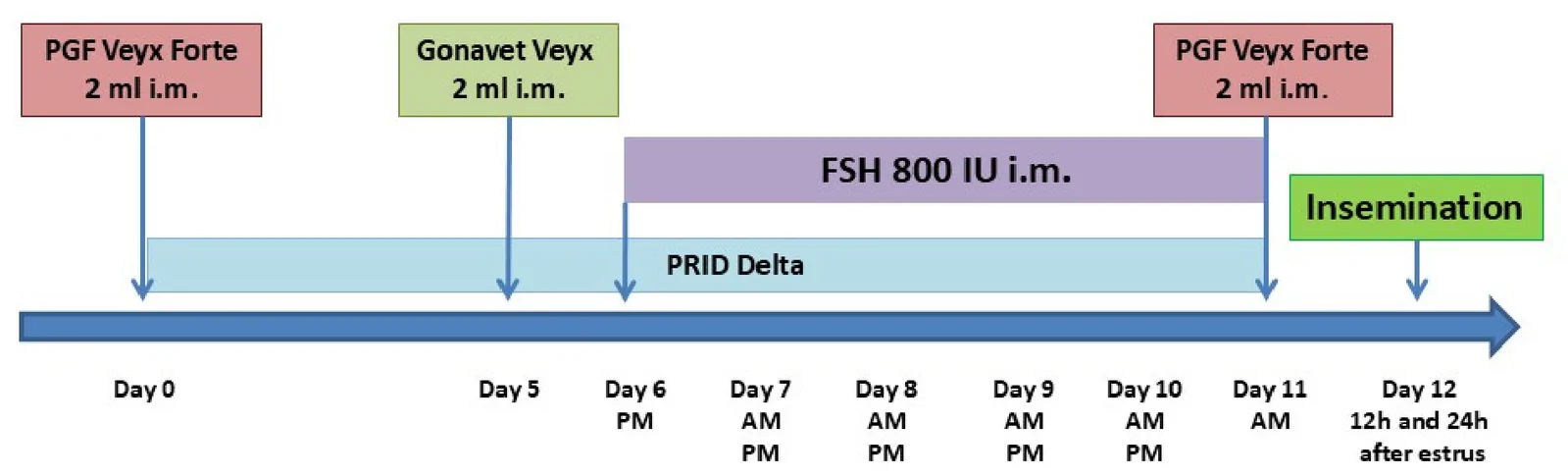
Estrus synchronization and superovulation of donor heifers.

On Day 0, 2 mL of PGF Veyx Forte (prostaglandin F2α; 250 μg/mL cloprostenol, Veyx-Pharma GmbH, Söhreweg, Schwarzenborn, Germany) was administered intramuscularly, and an intravaginal PRID delta (progesterone-releasing intravaginal device) (1.55 g of progesterone, Ceva Santé Animale, Z.I. Très le Bois, Loudéac, France) was inserted. On Day 5, 2 mL of Gonavet Veyx [6-D-Phe] (0.05 mg/mL gonadorelin, Veyx-Pharma GmbH, Söhreweg, Schwarzenborn, Germany) was administered intramuscularly. Superovulation was initiated on Day 6 with a total dose of 800 IU of follicle-stimulating hormone = FSH (Pluset, containing porcine follicle-stimulating hormone (p-FSH) and porcine luteinizing hormone (p-LH), typically 50 IU FSH/mL and 50 IU LH/mL, C/Barcelonès, 26, El Ramassar, Les Franqueses del Vallès, Barcelona, Spain) administered intramuscularly over 5 days at 12 h intervals. On Day 11, the PRID device was removed concurrently with the final FSH dose and administration of 2 mL of PGF2α used to induce estrus. Donors were artificially inseminated 12 and 24 h after the standing estrus detection using commercial sex-sorted semen (Figure 1).

### 2.3. Embryo Collection and Evaluation

Embryos were collected on Day 7 after insemination. Epidural anesthesia was induced using 5 mL of lidocaine (LidoBel, 20.0 mg lidocaine hydrochloride monohydrate, Bela-Pharm GmbH & Co. KG, Lohner Straße 19, Vechta, Germany). Both uterine horns were flushed with a pre-warmed flushing medium (EmXcell, IMV Technologies, L’Aigle, France) and embryos were recovered using a three-way silicone Foley catheter (IMV Technologies, L’Aigle, France). The recovered fluid was filtered and transferred to Petri dishes, and embryos were evaluated under a stereomicroscope (50× magnification) according to the International Embryo Technology Society guidelines [20], where stage 4 corresponds to morula, stage 5 to early blastocyst, stage 6 to blastocyst, and stage 7 to expanded blastocyst. Embryos were graded morphologically and maintained in holding medium at 37.5 °C. Only Grade 1 embryos at developmental stages 4–7 (morula to expanded blastocyst) were selected for fresh transfer or cryopreservation. Fresh embryos were transferred within 6 h of evaluation. Allocation to fresh transfer or freezing was determined by monthly operational recipient availability rather than morphological sorting: when the yield of fresh Grade 1 embryos exceeded the number of confirmed fit recipients on a given transfer day, surplus Grade 1 embryos were cryopreserved. Conversely, when qualified recipients exceeded fresh embryo supply, frozen–thawed Grade 1 embryos were transferred to utilize recipient capacity.

### 2.4. Embryo Cryopreservation and Thawing

Grade 1 embryos were cryopreserved using a conventional slow-freezing protocol with ethylene glycol and sucrose-based cryoprotectant following the manufacturer’s instructions. Freezing was performed using a programmable freezer (Embryo Freezer Ref. 027502: CL2200, IMV Technologies, L’Aigle, France). Frozen embryos cryopreserved in ethylene glycol for direct transfer were removed from liquid nitrogen and held in air for approximately 5–10 s. The straws were then immersed in a 30–35 °C water bath for 20–30 s until completely thawed. After thawing, each straw was carefully dried with a paper towel, loaded into a pre-warmed embryo transfer gun, and transferred directly into the synchronized recipient. Embryo transfer was performed as soon as possible after thawing, generally within 10–15 min, to maintain optimal embryo viability.

### 2.5. Recipient Synchronization and Embryo Transfer

**Figure 2:**
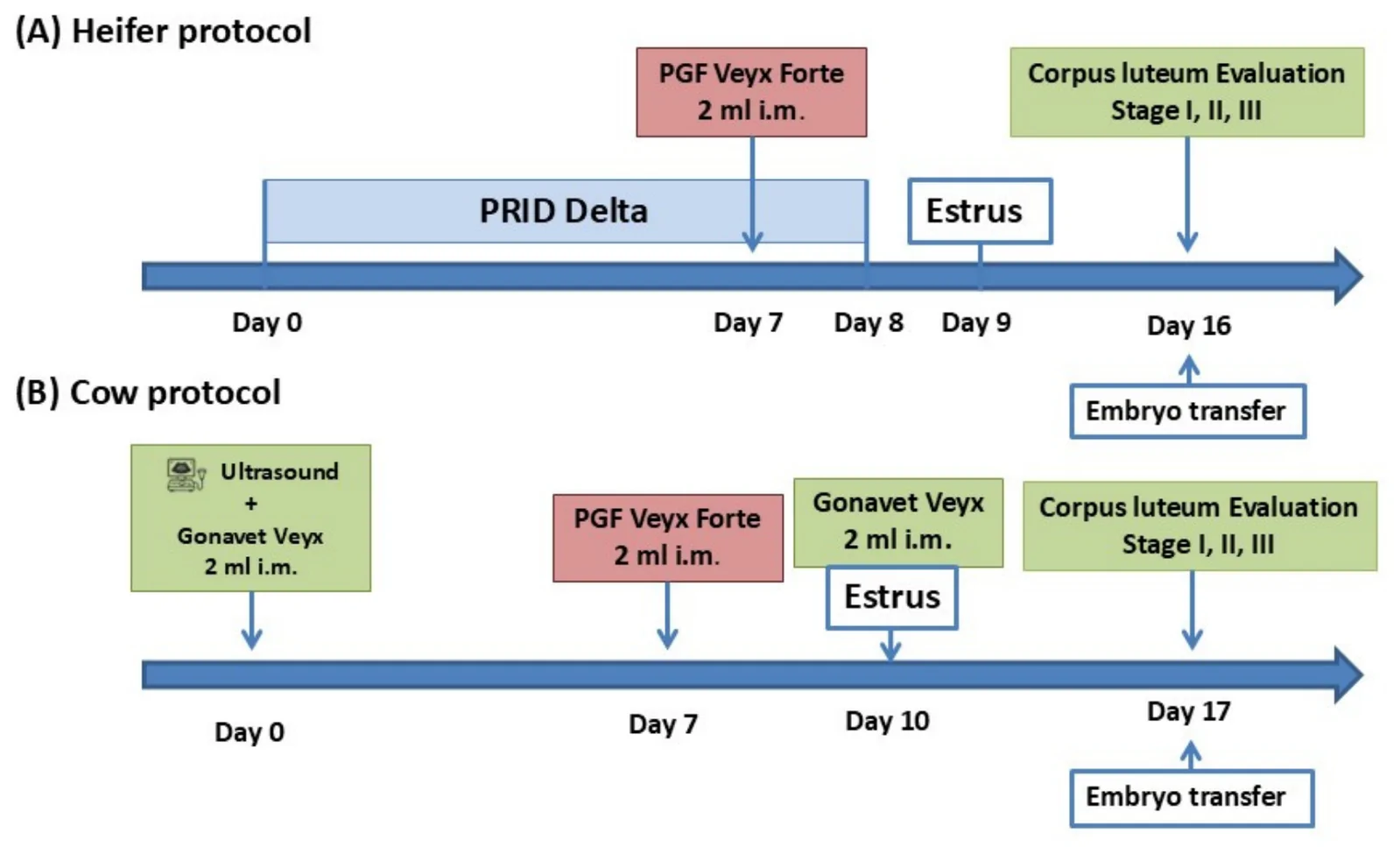
Estrus synchronization schedule of recipient heifer and cow for embryo transfer.

A total of 604 recipient animals (384 heifers and 220 cows) were synchronized and used for embryo transfer. Prior to embryo transfer, all recipient heifers and cows underwent a thorough gynecological examination via transrectal palpation and ultrasonography. Only animals with a normal, healthy uterus and a fully functional corpus luteum on one of the ovaries were selected for transfer. In total, 286 fresh and 318 frozen–thawed Grade 1 embryos were transferred. Heifers were synchronized using a progesterone-releasing intravaginal device (PRID delta) inserted on Day 0, followed by administration of PGF Veyx Forte (prostaglandin F2α; 250 μg/mL cloprostenol, Veyx-Pharma GmbH, Söhreweg, Schwarzenborn, Germany) on Day 7. The device was removed on Day 8 to induce estrus. A week later, the developmental status of the corpus luteum (stage I, II, III) was assessed rectally on Day 7 after estrus and classified as stage I, II, or III according to Basavalingappa et al. (2013) [21] based on size, consistency, and firmness, after which the embryo was transferred. In cows, synchronization began on Days 35–49 postpartum. Animals received Gonavet Veyx (0.05 mg/mL gonadorelin, Veyx-Pharma GmbH, Söhreweg, Schwarzenborn, Germany) followed 7 days later by PGF2α, and a second gonadorelin treatment on Day 10. Estrus was monitored and recorded. Embryos were transferred non-surgically into the uterine horn ipsilateral to the CL using an intrauterine transfer device (Figure 2). Embryo transfer was performed in recipient cows between 52 and 66 days in milk (DIM).

### 2.6. Pregnancy Diagnosis and Monitoring

Pregnancy was diagnosed by transrectal ultrasonography (Easi-Scan, IMV Imaging, Bellshill, UK) 34 days post-embryo transfer by a qualified veterinarian. Confirmed pregnant animals were subsequently monitored through parturition. Animals that were diagnosed as pregnant at Day 34 but failed to produce a live calf were classified as having experienced post-diagnosis gestation loss. Because continuous serial ultrasonographic examinations were not conducted between the initial pregnancy diagnosis and calving, the exact timing and physiological mechanism (e.g., resorption vs. late abortion) of gestation loss could not be definitively determined. Following non-pregnancy diagnosis or pregnancy loss, recipient heifers were re-entered into the artificial insemination program according to standard commercial herd management protocols, whereas multiparous cows were returned to the breeding herd for re-insemination up to 250 days in milk. Pregnancy rate per embryo transfer (PR/ET) was the proportion of total embryo transfers (*N* = 604) that resulted in a confirmed pregnancy at Days 30–35 via transrectal ultrasonography while calving proportion among pregnancies (CPP) was defined as the proportion of confirmed pregnant recipients (*N* = 315) that successfully carried the pregnancy to full-term calving.

### 2.7. Calf Management

Because sex-sorted semen yields predominantly female calves (~90% female purity), and to prevent sex bias in growth models, only female calves were included in the evaluation of birth weight, weaning weight, and pre-weaning weight gain. A total of 171 calves were born from the embryo transfers. During the experimental period, 3 calves died, resulting in a final study population of 168 calves at weaning. Calvings were routinely monitored by farm personnel, and no clinical dystocia or peri-parturient complications were observed across groups. Birth weights were recorded using a calibrated livestock scale (AM02-da-22). Each calf received 4 liters of high-quality colostrum (>25 Brix%) within 1 h after birth. Calves were housed individually and fed pasteurized whole milk (2 × 3.5 L), calf muesli, calf starter, and clean drinking water ad libitum. Animals were monitored until weaning, and data on growth performance, health status, and weaning weight were recorded. Milk feeding was continued until 80 days of age. Calves were weaned at 90 days of age according to the farm’s age-based weaning protocol rather than a body weight threshold.

Average daily gain (ADG) during the pre-weaning period was calculated using the following equation:

ADG(g/day)=[(WW−BW)×1000]/AW

where ***WW*** is the weaning weight (kg), ***BW*** is the birth weight (kg), and ***AW*** is the age at weaning (days). Since all calves were weaned at 90 days of age, ***AW*** was 90 days for all animals.

### 2.8. Statistical Analysis

Statistical analyses were performed using IBM SPSS Statistics (version 26.0; IBM Corp., Armonk, NY, USA). Pregnancy and calving rates were assessed using contingency tables, and differences among groups (embryo type, developmental stage, recipient category, and CL stage) were evaluated using the Chi-square test; Fisher’s exact test was applied when expected cell counts were <5. Following pregnancy diagnosis, calving outcomes were assessed using the same approach. The 95% confidence intervals (95% CI) for individual proportions were calculated using the Wilson score method. To evaluate the independent effects and potential confounding factors, multivariable logistic regression models were fitted including recipient category, CL stage, embryo status, and embryo developmental stage as independent variables. Odds ratios (ORs) and 95% confidence intervals (CIs) were calculated relative to designated reference categories. Continuous calf performance traits (birth weight, weaning weight, and pre-weaning weight gain) were tested for normality using the Shapiro–Wilk test and for homogeneity of variances using Levene’s test. Residual normality was also assessed using Q–Q plots. Residuals for most traits approximated a normal distribution; minor deviations were observed for birth weight, but these were considered acceptable given the sample size. Due to unbalanced commercial field data, each performance trait was analyzed using a univariate general linear model using Type III sums of squares. Fixed main effects included embryo type (fresh vs. frozen–thawed), embryo developmental stage (stages 4–7), recipient category (heifer vs. cow), and CL stage (I, II, III). Two-way interaction terms were evaluated and removed from the final models as they were not statistically significant (*p* ≥ 0.05) and did not improve overall model fit. Data are presented as Estimated Marginal Means ± standard error (SE) alongside 95% confidence intervals (95% CIs). Multiple comparisons were adjusted using Tukey’s post hoc test when variances were equal, or the Games–Howell test when unequal variance occurred. Statistical significance was defined as *p* < 0.05 and trends were declared at 0.05 ≤ *p* < 0.10.

## 3. Results

### 3.1. Pregnancy Rates per Embryo Transfer

#### 3.1.1. Univariate Analysis of Pregnancy Rates

A total of 604 embryo transfers were performed, including 384 in heifers and 220 in cows. The overall PR/ET was significantly higher in heifers, at 65.1% (250/384), than in cows, at 32.3% (71/220; *p* < 0.001; Table 1).

Recipient CL stage significantly affected pregnancy outcomes. PR/ET were 44.8% (99/221), 57.3% (215/375), and 87.5% (7/8) for CL stages I, II and III, respectively. Post hoc-adjusted residual analysis demonstrated that stage II resulted in a significantly higher PR/ET compared to stage I (*p* = 0.009 vs. *p* = 0.002), whereas differences involving stage III were not statistically significant (*p* > 0.017; Table 1). Due to the limited group size (*n* = 8), the PR/ET for CL stage III is presented as a descriptive observation with a wide 95% confidence interval (87.5%, 95% CI: 52.9–97.8%; Wilson score method), reflecting high statistical uncertainty.

There were 286 fresh and 318 frozen–thawed embryos transferred into 604 recipient heifers and cows. Fresh embryos achieved a higher PR/ET, at 62.9% (180/286), than frozen–thawed embryos, at 44.3% (141/318; *p* < 0.001; Table 1).

The effect of embryo developmental stage on PR/ET was also evaluated. Embryo developmental stage (stages 4–7) did not significantly influence pregnancy rate (*p* = 0.941). PR/ET were 55% (159/289), 50.8% (64/126), 51.7% (61/118), and 52.1% (37/71) for stages 4, 5, 6, and 7, respectively (Table 1).

#### 3.1.2. Multivariate Analysis of Pregnancy Rates

To evaluate the independent effects of recipient and embryo factors while controlling for potential confounding, a multivariable logistic regression analysis was conducted (Table 2). The overall model confirmed that recipient category and CL stage were the primary independent predictors of initial pregnancy success. Heifers achieved a significantly higher PR/ET than adult cows (*p* < 0.001), with cows having 77.6% lower odds of conception (OR = 0.224, 95% CI: 0.138–0.364). Regarding CL stage, recipients bearing a stage II CL had significantly greater odds of becoming pregnant compared to those with a stage I CL (OR = 1.639, 95% CI: 1.150–2.336, *p* = 0.006). Stage III CL recipients exhibited a high point-estimate odds ratio (OR = 8.116), though this failed to reach statistical significance (*p* = 0.060), likely due to the small sample size in this category. Notably, after adjusting for recipient parity and CL stage, embryo preservation status (fresh vs. frozen–thawed; *p* = 0.398) and embryo developmental stage (stages 4–7; *p* = 0.999) showed no significant independent association with initial pregnancy establishment.

### 3.2. Calving Proportion Among Pregnancies

#### 3.2.1. Univariate Analysis of Calving Proportion Among Pregnancies

Complete calving records were available for 248 of the 250 pregnant heifers and 67 of the 71 pregnant cows. The remaining six pregnancies (two heifers and four cows) were lost due to the death of the animals prior to term. Consequently, calving parameters were evaluated using these 248 heifers and 67 cows as denominators.

In addition to pregnancy outcomes, pregnancy maintenance to term was also assessed as a function of various factors. CPP tended to be higher in heifers than in cows, although the difference was not statistically significant (*p* = 0.078; Table 3). CPPs were 56.9% (141/248) in heifers and 44.8% (30/67) in cows, corresponding to embryo loss rates of 43.1% and 55.22%, respectively.

Among the influencing factors, we examined the effect of the CL at the time of embryo transfer on CPP. CL stage did not significantly affect CPP (*p* = 0.385). CPPs were 54.6% (53/97) for stage I and 55.0% (116/211) for stage II. Although stage III showed a lower CPP of 28.6% (2/7), interpretation is limited by the small sample size (Table 3).

The effect of fresh versus frozen–thawed embryos on CPP was also analyzed. Embryo type had no significant effect on CPP (*p* = 0.805), as CPPs were 53.7% (95/177) for fresh embryos and 55.1% (76/138) for frozen–thawed embryos (Table 3).

The developmental stage of the transferred embryos was also evaluated. All embryos were Grade 1 and classified as developmental stages 4–7. Embryo developmental stage did not have a statistically significant effect on pregnancy maintenance to term (*p* = 0.091), although a tendency was observed. CPPs were 48.7% (75/154), 60.3% (38/63), 65.6% (40/61), and 48.6% (18/37) for stages 4, 5, 6, and 7, respectively, with stage 6 embryos showing the highest survival (Table 3).

#### 3.2.2. Multivariate Analysis of Calving Proportion Among Pregnancies

The multivariable logistic regression model for CPP revealed distinct physiological patterns compared to initial pregnancy establishment (Table 4). Recipient category remained a significant independent factor (*p* = 0.012), with cows exhibiting significantly lower odds of successful calving than heifers (OR = 0.409, 95% CI: 0.204–0.819). In contrast to initial pregnancy establishment, initial Day 7 CL stage was no longer significantly associated with term calving rate (*p* = 0.508), indicating that early CL morphology does not dictate long-term gestation maintenance once pregnancy is established. Crucially, embryo-related factors demonstrated a more prominent role in term calving success. Embryos transferred at stage 6 yielded a significantly higher CPP compared to stage 4 (OR = 2.035, 95% CI: 1.089–3.803, *p* = 0.026), with stage 5 showing a similar positive trend (OR = 1.793, *p* = 0.061). Furthermore, the transfer of fresh embryos displayed a strong statistical trend toward improved calving rates compared to frozen–thawed embryos (OR = 1.770, 95% CI: 0.990–3.164, *p* = 0.054).

### 3.3. Calf Performance

The effects of embryo type, recipient CL stage, recipient category, and embryo developmental stage on calf performance, as assessed by birth weight, weaning weight, and pre-weaning average daily weight gain, are summarized in Table 5. Embryo type significantly affected birth weight (*p* = 0.004), with heifer calves derived from frozen–thawed embryos being heavier at birth than those from fresh embryos (37.16 ± 1.16 vs. 39.26 ± 1.07 kg). However, embryo type had no effect on weaning weight (*p* = 0.929) or pre-weaning growth (*p* = 0.439). Recipient CL stage did not significantly influence any of the evaluated parameters (birth weight: *p* = 0.324; weaning weight: *p* = 0.786; weight gain: *p* = 0.879). Although numerically higher mean values were observed for individuals in stage III, the very small sample size of this group limited interpretation. Similarly, recipient category (heifer vs. cow) had no significant effect on calf performance traits (*p* = 0.059, 0.688 and 0.376, respectively). Although a slightly higher initial body weight and body weight gain were observed in cows, these differences were not statistically significant. Embryo developmental stage also showed no significant associations with birth weight (*p* = 0.235), weaning weight (*p* = 0.820), or pre-weaning weight gain (*p* = 0.740), despite minor numerical differences among groups.

## 4. Discussion

This study evaluated key factors affecting embryo transfer success outcomes in Holstein–Friesian cattle, with particular emphasis on pregnancy establishment, pregnancy maintenance to term, and calf growth parameters. The results demonstrate that both recipient- and embryo-related factors influence different stages of the reproductive process and term calving success to varying extents.

In field-based embryo transfer programs, single-factor comparisons can be confounded by non-random embryo allocation (e.g., technicians preferring to transfer fresh or higher-grade embryos into young heifers rather than multiparous cows). Because each transfer in our study was performed into a separate, unique recipient animal, evaluating outcomes through multivariable logistic regression allowed us to isolate the true independent effect of each variable while controlling for these confounding group allocations. By controlling for these variables simultaneously, our models revealed distinct physiological mechanisms governing initial pregnancy establishment versus full-term calving success. Furthermore, because pregnancy was diagnosed at Day 34 post-transfer and recipients were subsequently monitored through parturition without sequential ultrasonography, animals failing to calve were classified strictly as experiencing post-diagnosis gestation loss. Without serial ultrasound checks, attributing these losses to specific gestational windows (e.g., early embryonic resorption versus late fetal abortion) remains speculative under commercial field conditions.

Across both unadjusted and multivariable models, the most prominent finding was the significantly higher reproductive performance in heifers compared to multiparous cows. Confirmed CPPs in heifers were more than double those of cows, aligning with well-documented differences in metabolic clearance of progesterone, uterine environment quality, and lactation-induced metabolic stress in high-yielding dairy cows [22,23,24]. These findings further highlight the imperative of prioritizing heifer recipients in commercial embryo transfer programs.

A stage II CL significantly enhanced initial PR/ET, confirming that optimal early luteal function and progesterone secretion on Day 7 are crucial for maternal recognition of pregnancy. However, CL morphology on Day 7 was no longer predictive of term calving success. This suggests that once embryonic implantation and early placental attachment are successfully achieved, initial morphological differences in the CL do not limit long-term pregnancy survival. While embryo stage showed no significant impact on initial 30-day pregnancy status, transfer of stage 6 significantly increased the odds of term calving compared to stage 4. Furthermore, fresh embryos demonstrated a strong statistical trend toward higher calving success than frozen–thawed embryos. This indicates that subtle cryopreservation-induced organelle damage or developmental lag at the morula stage may not prevent initial pregnancy detection at Days 30–35, but significantly increases the risk of late embryonic or fetal mortality during mid-to-late gestation.

One of the most prominent findings was the significantly higher PR/ET in heifers compared to cows. The PR/ET observed in heifers (65.1%) was more than double that of cows (32.3%), confirming previous reports of superior reproductive performance in heifers [22,23]. This difference is most likely attributable to a more favorable uterine environment, lower metabolic load, and the absence of lactation. In contrast, lactating cows often experience negative energy balance and associated endocrine alterations, which may impact early embryo–maternal communication during the critical period of pregnancy establishment [24]. These findings further highlight the importance of appropriate recipient selection in embryo transfer programs.

Recipient CL stage also influenced Day 30–35 pregnancy outcomes. Recipients with stage II CL showed significantly higher PR/ET than those with stage I CL, which aligns with the central role of progesterone in preparing the endometrium for implantation. Lower progesterone levels during the early luteal phase may compromise uterine receptivity and reduce the likelihood of successful implantation [25]. Although the highest PR/ET was observed in stage III CL, this finding must be interpreted with substantial caution. Because only eight recipients were classified as stage III CL, the resulting 95% confidence interval was extremely wide, reflecting high statistical uncertainty due to the limited sample size. Consequently, this observation remains descriptive and precludes definitive conclusions regarding the statistical superiority of stage III CL recipients.

Embryo type had a significant effect on the PR/ET, with fresh embryos outperforming frozen–thawed embryos. This result is consistent with the well-documented negative impact of cryopreservation-induced cellular damage, osmotic stress, and molecular alterations on embryo viability [26,27]. However, embryo type did not significantly affect calving rate, suggesting that embryos capable of establishing pregnancy have comparable developmental potential regardless of origin. This indicates that the primary impact of cryopreservation occurs during the early stages of pregnancy.

Embryo developmental stage (within stages 4–7) did not influence PR/ET, indicating similar implantation potential across these stages. Furthermore, embryo developmental stage did not significantly affect CPP, although a non-significant numerical tendency toward higher CPP was observed in stage 6 embryos. While this numerical trend could have suggested optimal synchrony between embryo development and uterine receptivity, it did not reach statistical significance. Previous studies have reported inconsistent findings regarding the optimal developmental stage [28,29,30,31,32], which likely reflects differences in management conditions and synchronization protocols.

Post-diagnosis gestation loss remained a major limiting factor for reproductive efficiency, particularly in cows, where higher loss rates were observed. This supports earlier findings linking increased post-diagnosis loss to metabolic and physiological stress in high-producing dairy cows [24]. In contrast, neither CL stage nor embryo type significantly affected CPP, suggesting that once pregnancy is established, other factors—such as maternal immune function, placental development, and environmental conditions—may play a more dominant role in maintaining pregnancy.

With respect to calf performance, embryo type significantly affected birth weight, with calves derived from frozen–thawed embryos being heavier at birth than those from fresh embryos. This finding should be interpreted with caution, though it is consistent with previous studies suggesting that cryopreservation may induce epigenetic or developmental alterations influencing fetal growth [33,34,35]. The observed increase (2.1 kg) exceeds the magnitude typically reported in the literature, where differences are generally modest [36]. Because our study utilized exclusively in vivo-derived embryos, extrapolating cellular or epigenetic mechanisms remains speculative. Furthermore, potential unmeasured confounders under commercial field conditions—such as subtle variations in gestation length, dam parity, sire genetic potential, and seasonal nutrition—could readily account for a 2.1 kg variation at birth. Importantly, these differences did not persist until weaning, which may demonstrate that initial birth weight variations did not impede overall pre-weaning growth. Therefore, these data demonstrate a minor statistical association with birth weight in heifer calves rather than a direct mechanistic effect of cryopreservation on fetal growth. Neither recipient category, CL stage, nor embryo developmental stage significantly influenced growth traits, suggesting that these factors have limited long-term impact on postnatal performance under the conditions studied.

Certain limitations of this study should be acknowledged. CL status was evaluated on Day 7 via rectal palpation under commercial farm conditions, rather than utilizing color Doppler ultrasonography to quantify luteal blood flow and functional vascularization. While ultrasound provides greater precision, rectal palpation reflects the standard practical field methodology across commercial MOET setups. The interpretation of certain findings (e.g., CL stage III) is limited by the small sample size. In addition, as the study was conducted on a single commercial farm, the generalizability of the results may have been limited. To our knowledge, this is one of the few studies evaluating embryo type, developmental stage, recipient category, and CL stage simultaneously under commercial farm conditions. From a practical perspective, these findings support the view that prioritizing heifer recipients and the use of fresh embryos when maximizing PR/ET is the primary objective.

## 5. Conclusions

Overall, the results indicate that recipient selection—particularly the use of heifers—and embryo type are key determinants of embryo transfer success, with fresh embryos yielding a higher Day 30–35 pregnancy rate than frozen–thawed embryos. Embryo developmental stage did not significantly affect either initial pregnancy rates or full-term calving success, although stage 6 embryos exhibited a non-significant numerical trend toward higher calving rates. Postnatal performance was largely independent of embryo type and recipient factors; although a minor positive association with birth weight was observed for frozen–thawed transfers among live-born heifer calves, this difference did not translate into altered growth rates or body weight at weaning. These findings highlight that optimizing pregnancy outcomes and postnatal performance involves distinct biological processes. From a practical perspective, prioritizing heifer recipients and fresh embryos may improve reproductive efficiency under commercial conditions.

## Figures and Tables

**Table 1:** Univariate analysis of factors influencing initial pregnancy rates in recipient cattle.

Factor	Category	*n*/*N*	PR/ET (%)	95% CI ^†^	χ^2^	df	*p*-Value
Recipient category	Heifer	250/384	65.10% ^a^	60.2–69.7%	60.55	1	<0.001
	Cow	71/220	32.30% ^b^	26.4–38.8%			
CL stages	I	99/221	44.80% ^b^	38.4–51.4%	12.62	2	0.002
	II	215/375	57.30% ^a^	52.3–62.2%			
	III	7/8	87.50% ^ab^	52.9–97.8%			
Embryo type	Fresh	180/286	62.90% ^a^	57.2–68.3%	20.91	1	<0.001
	Frozen–thawed	141/318	44.30% ^b^	39.0–49.8%			
Developmental stage	Stage 4	159/289	55.00%	49.2–60.7%	0.82	3	0.941
	Stage 5	64/126	50.80%	42.2–59.3%			
	Stage 6	61/118	51.70%	42.7–60.6%			
	Stage 7	37/71	52.10%	40.6–63.3%			

^†^ The 95% confidence intervals for proportions are calculated using the Wilson score method. ^a,b^ Percentages within the same factor sharing different superscript letters differ significantly based on post hoc-adjusted residual analysis with Bonferroni correction (*p* < 0.017).

**Table 2:** Multivariable logistic regression analysis for factors influencing pregnancy rate in recipient cattle.

Factor	Category	B	SE	Wald	*p*-Value	Odds Ratio (OR)	CI for OR
Embryo type	Fresh	Reference	-	-	-	1.000	-
	Frozen–thawed	0.199	0.236	0.714	0.398	1.221	0.769–1.938
CL stage	I	Reference	-	-	0.006	1.000	-
	II	0.494	0.181	7.474	0.006	1.639	1.150–2.336
	III	2.094	1.113	3.541	0.060	8.116	0.917–71.861
Recipient category	Heifer	Reference	-	-	-	1.000	-
	Cow	−1.496	0.248	36.428	< 0.001	0.224	0.138–0.364
Developmental stage	Stage 4	Reference	-	-	0.999	1.000	-
	Stage 5	−0.012	0.230	0.003	0.959	0.988	0.630–1.551
	Stage 6	−0.004	0.234	0.000	0.987	0.996	0.630–1.575
	Stage 7	0.039	0.288	0.018	0.893	1.039	0.591–1.828

CI: Confidence interval.

**Table 3:** Univariate analysis of factors influencing full-term calving success in recipient cattle.

Factor	Category	*n*/*N*	CPP (%)	95% CI ^†^	χ^2^	df	*p*-Value
Recipient category	Heifer	141/248	56.90%	50.7–62.9%	3.1	1	0.078
	Cow	30/67	44.80%	33.4–56.7%			
CL stages	I	53/97	54.60%	44.7–64.2%	1.91	2	0.385
	II	116/211	55.00%	48.3–61.5%			
	III	2/7	28.60%	8.2–64.1%			
Embryo type	Fresh	95/177	53.70%	46.3–60.9%	0.06	1	0.805
	Frozen–thawed	76/138	55.10%	46.7–63.1%			
Developmental stage	Stage 4	75/154	48.70%	40.9–56.6%	6.47	3	0.091
	Stage 5	38/63	60.30%	47.8–71.6%			
	Stage 6	40/61	65.60%	53.1–76.2%			
	Stage 7	18/37	48.60%	33.5–64.1%			

^†^ The 95% confidence intervals for proportions are calculated using the Wilson score method.

**Table 4:** Multivariable logistic regression analysis for factors influencing gestation maintenance and calving outcomes in recipient cattle.

Factor	Category	B	SE	Wald	*p*-Value	Odds Ratio (OR)	CI for OR
Embryo type	Fresh	Reference	-	-	-	1.000	-
	Frozen–thawed	0.571	0.297	3.705	0.054	1.770	0.990–3.164
CL stage	I	Reference	-	-	0.508	1.000	-
	II	0.053	0.253	0.045	0.833	1.055	0.642–1.733
	III	−0.944	0.872	1.171	0.279	0.389	0.070–2.151
Recipient category	Heifer	Reference	-	-	-	1.000	-
	Cow	−0.893	0.354	6.362	0.012	0.409	0.204–0.819
Developmental stage	Stage 4	Reference	-	-	0.069	1.000	-
	Stage 5	0.584	0.312	3.508	0.061	1.793	0.973–3.305
	Stage 6	0.711	0.319	4.965	0.026	2.035	1.089–3.803
	Stage 7	0.027	0.374	0.005	0.941	1.028	0.494–2.139

CI: Confidence interval.

**Table 5:** Descriptives of evaluated traits *.

Factors	Birth Weight (kg)	Weaning Weight (kg)	Pre-Weaning Weight Gain (g/day)
Embryo type Fresh Frozen–thawed	*p* = 0.004	*p* = 0.929	*p* = 0.439
	37.16 ± 1.165 ^b^ [34.86–39.47] (*N* = 95)	128.78 ± 4.597 [119.70–137.85] (*N* = 92)	989.36 ± 44.215 [902.04–1076.68] (*N* = 92)
	39.26 ± 1.071 ^a^ [37.15–41.38] (*N* = 76)	128.52 ± 4.217 [120.19–136.85] (*N* = 76)	968.14 ± 40.561 [888.03–1048.24] (*N* = 76)
CL stage I	*p* = 0.342	*p* = 0.786	*p* = 0.857
	38.55 ± 0.682 [37.20–39.89] (*N* = 53)	125.89 ± 2.705 [120.54–131.23] (*N* = 52)	963.92 ± 26.019 [912.53–1015.30] (*N* = 52)
II III	37.57 ± 0.481 [36.62–38.52] (*N* = 116)	126.42 ± 1.902 [122.66–130.18] (*N* = 114)	957.94 ± 18.294 [921.81–994.07] (*N* = 114)
	38.52 ± 2.874 [32.85–44.20] (*N* = 2)	133.63 ± 11.312 [111.29–155.97] (*N* = 2)	1014.38 ± 108.812 [799.49–1229.27] (*N* = 2)
Recipient category	*p* = 0.059	*p* = 0.688	*p* = 0.376
Heifer	39.11 ± 0.996 [37.14–41.07] (*N* = 141)	127.91 ± 3.922 [120.16–135.65] (*N* = 138)	963.01 ± 37.722 [888.51–1037.50] (*N* = 138)
Cow	37.32 ± 1.301 [34.75–39.89] (*N* = 30)	129.39 ± 5.127 [119.26–139.51] (*N* = 30)	994.49 ± 49.313 [897.10–1091.87] (*N* = 30)
Developmental stage	*p* = 0.235	*p* = 0.820	*p* = 0.740
Stage 4	39.05 ± 1.045 [36.99–41.11] (*N* = 75)	129.76 ± 4.112 [121.63–137.88] (*N* = 75)	976.22 ± 39.554 [898.10–1054.33] (*N* = 75)
Stage 5	38.69 ± 1.181 [36.36–41.02] (*N* = 38)	127.16 ± 4.647 [117.98–136.34] (*N* = 38)	959.37 ± 44.704 [871.08–1047.65] (*N* = 38)
Stage 6	37.73 ± 1.205 [35.35–40.11] (*N* = 40)	129.87 ± 4.792 [120.41–139.34] (*N* = 37)	998.06 ± 46.096 [907.02–1089.10] (*N* = 37)
Stage 7	37.39 ± 1.431 [34.60–40.18] (*N* = 18)	127.80 ± 5.561 [116.82–138.78] (*N* = 18)	981.34 ± 53.589 [875.71–1086.97] (*N* = 18)

* Values represent the Estimated Marginal Means (least-squares means) ± standard error of the mean (SE) along with [95% confidence intervals] and sample size (N) derived from univariate general linear models. Means with different superscript lowercase letters within the same factor differ according to ANOVA (*p* < 0.05).

## Data Availability

The data presented in this study are available on request from the corresponding author. The data are not publicly available due to privacy restrictions.

## Abbreviations

The following abbreviations are used in this manuscript:

AI	Artificial insemination
ART	Assisted reproductive technology
CL	Corpus luteum
FSH	Follicle-stimulating hormone
MOET	Multiple ovulation and embryo transfer
